# Prospective Study of Engagement in Leisure Activities and All-Cause Mortality Among Older Japanese Adults

**DOI:** 10.2188/jea.JE20200427

**Published:** 2022-06-05

**Authors:** Takaki Kobayashi, Yukako Tani, Shiho Kino, Takeo Fujiwara, Katsunori Kondo, Ichiro Kawachi

**Affiliations:** 1 Department of Global Health Promotion, Tokyo Medical and Dental University, Tokyo, Japan; 2 Harvard T.H. Chan School of Public Health, Boston, Massachusetts, USA; 3 Department of Social Preventive Medical Sciences, Center for Preventive Medical Sciences, Chiba University, Chiba, Japan

**Keywords:** aging, leisure activities, Japan, older people

## Abstract

**Background:**

Engagement in leisure activities among older people is associated with a lower risk of mortality. However, no studies have been conducted focusing on the difference of associations with mortality risk among multiple types of leisure activities.

**Methods:**

We examined prospectively the association of engagement in leisure activities with all-cause mortality in a cohort of older Japanese adults. The Japan Gerontological Evaluation Study included 48,216 participants aged 65 years or older. During a mean follow-up period of 5.6 years, we observed 5,575 deaths (11.6%). We investigated the total number of leisure activities, as well as combinations of 25 different leisure activities with Cox proportional hazards models, adjusting for potential confounding factors.

**Results:**

We found a linear relationship between the total number of leisure activities and mortality hazard (adjusted hazard ratio, 0.93; 95% CI, 0.92–0.95). Furthermore, engagement in leisure activities involving physical activity, as well as group-based interactions, showed the strongest associations with lowered mortality. By contrast, engagement in cultural leisure activities and solitary leisure activities were not associated with all-cause mortality.

**Conclusion:**

Although we cannot rule out residual confounding, our findings suggest that encouraging engagement in physically-active group-based leisure activities may promote longevity in older adults.

## INTRODUCTION

Leisure activities have been defined as “the voluntary use of free time for activities outside the daily routine”,^1^ and “activities that individuals engage in for enjoyment or well being which are independent of work or activities of daily living”.^2^ Engagement in leisure activities among older people is associated with a lower risk of mortality.^3^^–^^7^ There are several postulated mechanisms linking engagement in leisure activities to health. Most obviously, many leisure activities involve physical activity, and in turn, physical activity is associated with a lower risk of cardiovascular disease,^8^ cancer,^9^ diabetes,^10^ and cognitive decline.^1^^,^^11^ By contrast, sedentary behavior is associated with a higher risk of chronic diseases and mortality.^12^^–^^14^ However, engagement in some sedentary leisure activities—for example, cognitive activities and cultural activities—has been suggested to be associated with beneficial health outcomes.^1^^,^^15^^–^^20^

Participation in cognitively stimulating activities decreases the risk of cognitive impairment and dementia,^1^^,^^15^^,^^16^ and dementia is associated with a higher risk of mortality.^21^ A subset of cognitively stimulating activities, cultural engagement, also has been suggested to reduce the risk of depression^17^ and mortality,^18^^,^^19^ as well as cognitive decline.^20^ Finally, some leisure activities are inherently social in nature because they are conducted in groups. Engagement in social activity is also associated with many health benefits. Previous studies showed that social participation lowered the risk of dementia,^1^^,^^22^ isolation,^23^ mental health problems,^24^ and mortality.^25^

In Japan, several studies have been conducted on the relationship between leisure activities and mortality, but the measurements of leisure activities are limited. That is, previous studies using the Japanese sample did not measure the specific leisure activities and failed to count the total number of leisure activities.^6^^,^^26^ A study among 1,853 older adults residing in one prefecture of Japan only assessed whether they have any hobbies.^6^ Another study among 3,583 older adults residing in one Japanese prefecture focused only on the structural aspects of hobbies, such as physical or cultural, and solitary or group activities.^26^ Furthermore, these studies showed inconsistent findings; the former study reported engagement in leisure activities was associated with a lower risk of mortality,^6^ whereas the latter study did not detect a significant relationship between them.^26^ One reason for the inconsistent results may be that the total number of leisure activities was not evaluated. A study among Swedish older adults, in which the majority of older adults had multiple leisure activities, found a dose-response association between the total number of leisure activities and mortality.^5^ Thus, the association between the total number of leisure activities and mortality among Japanese older people needs investigation.

Although there are some studies from western countries that reported the link between leisure activities and mortality,^3^^–^^5^ investigations are needed in Japan because the popular types of leisure activities differ between Japan and other countries. For example, reading books was the most common leisure activity among Swedish older adults,^5^ and physical activities have been reported to be popular among older adults in the United States,^27^ while gardening was the most common among Japanese older adults.^28^ The variety may result from cultural differences, which raises the rationale for further study in Japan. Moreover, to our knowledge, there are no large-scale cohort studies conducted in Japan about the association between engagement in leisure activities and all-cause mortality. Furthermore, no studies have been conducted focusing on the difference of associations with mortality risk among multiple types of leisure activities.

The present study investigated the association between the total number of leisure activities and mortality. Further, we aimed at examining different types of leisure activities that may have diverse mechanisms toward affecting the risk of mortality. Our primary study hypothesis is that the more leisure activities in which an individual engages, the better their chance of living longer. The reason is that engaging in a broader range of activities increases the probability of being exposed to a wider range of health benefits (eg, physical activity, social interaction, and cognitive stimulation). At the same time, some types of leisure activity (such as playing pachinko—a popular type of gambling in Japan, similar to slot machines) are likely to be less healthy, because of prolonged sedentarism and exposure to secondhand smoke and noise. Hence, we sought to examine leisure activities according to the different mechanisms likely to be involved, such as physical activity, social participation, cultural engagement, and cognitive stimulation.

## METHODS

### Study participants

The Japan Gerontological Evaluation Study (JAGES) is a nationwide, population-based cohort study established in 2010 to investigate the social determinants of healthy aging among non-disabled community-dwelling residents aged 65 years or older.^29^^,^^30^ The baseline survey was conducted between August 2010 and January 2012. We distributed questionnaires to 95,827 community-dwelling older adults, in 13 municipalities, throughout Japan. The response rate was 65.1% (*n* = 62,426) among which 56,687 had valid information on sex and age. Of 56,687 valid participants, a total of 54,537 (96.2%) participants were successfully followed for a period of 6 years. The analytic sample for the present study consists of 48,216 participants (22,178 men and 26,038 women), after exclusion of participants who were missing information about their leisure activities (*n* = 1,938), those who reported significant disability in activities of daily living, defined as being unable to walk, take a bath, or use the toilet without assistance (*n* = 2,476), and those who were missing answers to 7 or more of the 13 questions measuring instrumental activities of daily living (*n* = 2,422). Participants were informed that participation in the study was voluntary and that returning the questionnaire indicated their consent to participate in the study. The protocol of this study was approved by the Ethics Committees of the National Center for Geriatrics and Gerontology (No. 992-3).

### Outcome variable

Our primary outcome was mortality. We retrieved information on mortality from 2010 to 2016 from the government database of the public long-term care insurance system. Among these records, there were 5,575 (11.6%) deaths identified in the analytic sample. Those who moved out of the municipalities (2,150 out of 56,687, 3.8%) were censored on the date of its notification at the municipal offices.

### Explanatory variable

We evaluated participants’ engagement in leisure activities by using the following two questions, “Do you have any hobbies or take lessons? (Yes/No),” and “Which of the following are your hobbies or lessons? (mark all that apply): golf, mini golf, gate ball, exercise/Tai Chi, walking/jogging, Go/Shogi/Mahjong, reading, personal computer [PC] use, playing musical instruments, chorus/folk song, karaoke, dancing, Haiku/Tanka/Senryu, calligraphy, tea ceremony/flower arrangement, craft, painting/hand-drawn postcards, photography, gardening, growing crops, traveling, hiking, fishing, pachinko, and other.” Among the free descriptive answers to leisure activities conducted in the previous JAGES survey in 2006, we selected 25 leisure activities that were often described by participants. Leisure activities with the same activity content but different activity names were combined as an option. We excluded individuals with missing answers on the baseline survey. We then constructed several alternative ways of summarizing leisure activities. For the main analyses, we defined the total number of leisure activities based on the responses: “0,” “1,” “2,” “3,” “4,” “5,” and “6 or more”.^5^ Participants who engaged in 7 or more leisure activities accounted for only 3.4% of the sample, thus we combined this group with the participants who engaged in 6 leisure activities, to make a “6 or more” grouping.

For secondary analyses, we grouped the 25 leisure activities in two ways. First, we grouped the activities based on whether they involved predominantly “physically-active leisure activities (golf, mini golf, gate ball, exercise/Tai Chi, walking/jogging, dancing, gardening, growing crops, and hiking),” “cultural leisure activities (Go/Shogi/Mahjong, reading, playing musical instruments, chorus/folk song, Haiku/Tanka/Senryu, calligraphy, tea ceremony/flower arrangement, craft, painting/hand-drawn postcards, and photography),” or “other leisure activities (PC, karaoke, traveling, fishing, pachinko, and other).”^4^^,^^15^^,^^22^^,^^26^^,^^31^^–^^33^

Second, we grouped the leisure activities according to whether they predominantly involved “group-based leisure activities (golf, mini golf, gate ball, Go/Shogi/Mahjong, chorus/folk song, karaoke, and dancing),” “solitary leisure activities (reading, PC, playing musical instruments, Haiku/Tanka/Senryu, calligraphy, craft, painting/hand-drawn postcards, fishing, and pachinko),” or “others (exercise/Tai Chi, walking/jogging, tea ceremony/flower arrangement, photography, gardening, growing crops, traveling, hiking, and other).”^15^^,^^22^^,^^26^^,^^31^^,^^34^^,^^35^

### Covariates

We adjusted for the following baseline variables as potential confounders of the relationship between engagement in leisure activities and mortality: sex, age, educational attainment, annual household income, employment status, living situation, marital status, smoking status, alcohol intake, body mass index, instrumental activities of daily living,^36^ depressive symptoms (defined using the short version of the Geriatric Depression Scale),^37^ cognitive complaints,^38^ self-rated health, and self-reported disease diagnosis (cancer, heart disease, stroke, diabetes mellitus, respiratory disease, and others).^39^^,^^40^ Moreover, we selected as potential mediating variables frequency of meeting friends,^41^ number of social interactions with friends/acquaintances,^42^ and social support (receiving).^40^^,^^42^ Multiple imputations were conducted for missing data on questions to measure instrumental activities of daily living. We imputed the missing values of 13 questions regarding instrumental activities of daily living by using all the other variables used in the present analyses. We used the “mi” command of STATA (Stata Corp, College Station, TX, USA) for multiple imputation through the Markov chain Monte Carlo method and created 20 imputed datasets.

### Statistical analysis

We used Cox proportional hazards models to evaluate the association of engagement in leisure activities with all-cause mortality. Model 1 adjusted for sex, age, and socioeconomic status. Model 2 additionally adjusted for the other potential confounding variables. Model 3 further adjusted for potential mediating factors; social network, and social support. The analyses were repeated by excluding the deaths occurring within the first 1, 2, and 3 years of follow-up in order to address reverse causality.

First, we assessed possible interaction by sex. When we added an interaction term between the total number of leisure activities (continuous) and sex (categorical) to the regression models, it was not statistically significant (*P* = 0.76). We also assessed possible interaction by age (categorical), and it was not statistically significant either (*P* > 0.5). Hence, we present all results combining both sex and all ages.

Besides, we computed the E-values to assess residual confounding. Although no threshold cutoff is proposed, E-values provide an assessment of how strongly an unmeasured confounding variable would need to be associated with the exposure and outcome in order to fully explain away the observed associations.^43^ Accordingly, larger E-values imply that substantial unmeasured confounding would be needed to explain away the observed association. E-value is calculated by using the observed hazard ratio [HR] of *HR*, and ${\text{HR}}^{⋆}$ = inverse of *HR*:

when HR>1, E-value=HR+sqrt{HR×(HR−1)},

$$\text{when }HR<1,\text{ E-value}={HR}^{⋆}+sqrt\{{HR}^{⋆}\times ({HR}^{⋆}-1)\}.$$

Two sets of sensitivity analyses were conducted with different classifications of leisure activities. First, we stratified the analysis by “physically-active leisure activities (golf, mini golf, gate ball, exercise/Tai Chi, walking/jogging, dancing, gardening, growing crops, and hiking),” “sedentary leisure activities (Go/Shogi/Mahjong, reading, PC, playing musical instruments, karaoke, Haiku/Tanka/Senryu, calligraphy, tea ceremony/flower arrangement, craft, painting/hand-drawn postcards, and pachinko),” and “the others (chorus/folk, photography, traveling, fishing, and other).”^12^^,^^14^ “Physically-active leisure activities” are the same as those of the earlier analysis, while “sedentary leisure activities” are slightly different from “cultural leisure activities.” For example, “PC,” “karaoke,” and “pachinko” were grouped under “sedentary leisure activities,” although they were not included in “cultural leisure activities” in the previous analysis. Secondly, we compared “physically-active group-based leisure activities (golf, mini golf, gate ball, and dancing),” “physically-active non-group-based leisure activities (exercise/Tai Chi, walking/jogging, gardening, growing crops, and hiking),” “non-physically-active non-solitary leisure activities (Go/Shogi/Mahjong, chorus/folk song, karaoke, tea ceremony/flower arrangement, photography, traveling, and other),” and “non-physically-active solitary leisure activities (reading, PC, playing musical instruments, Haiku/Tanka/Senryu, calligraphy, craft, painting/hand-drawn postcards, fishing, and pachinko).” The idea was that we would compare the four types of leisure activities, “physically-active group-based,” “physically-active non-group-based,” “non-physically-active group-based,” and “non-physically-active non-group-based” leisure activities in the light of the two analyses above.

Finally, we examined the association of each leisure activity with all-cause mortality (simultaneously mutually adjusted). All analyses were performed using Stata software (version 14.2) at a significance level of 0.05.

## RESULTS

Among the eligible 48,216 participants, 5,575 (11.6%) deaths occurred over a mean of 5.6 years of follow-up or 270,311 person-years. Table 1 shows the baseline characteristics of the participants, according to the reported total number of leisure activities. 28.9% of the population had “0” leisure activity, while 54.0% had two or more leisure activities. The total number of leisure activities varied based on socioeconomic status (ie, education, income, and employment status), instrumental activities of daily living, depression score, self-rated health, social network, and social support. For example, the total number of leisure activities is more likely to be larger among those with higher socioeconomic status, male gender, married status, no cognitive complaints, better self-rated health, being socially active, and receiving social supports. The baseline characteristics of male and female participants are shown in eTable 1. Among 22,178 male participants, 3,519 (15.9%) deaths occurred, and among 26,038 female participants, 2,056 (7.9%) deaths occurred over the follow-up period.

**Table 1.** Baseline characteristics of older Japanese participants (*n* = 48,216) who were 65 years of age or older, Japan, 2010–2016

Characteristic	Total number of leisure activities													

	0 (*n* = 13,953)		1 (*n* = 8,228)		2 (*n* = 8,197)		3 (*n* = 6,842)		4 (*n* = 4,711)		5 (*n* = 2,957)		6–17 (*n* = 3,328)	
						
	No.	%	No.	%	No.	%	No.	%	No.	%	No.	%	No.	%
Deaths	2,077	14.9	1,180	14.3	921	11.2	630	9.2	363	7.7	208	7.0	196	5.9
Sex														
Male	5,833	41.8	3,754	45.6	3,769	46.0	3,280	47.9	2,257	47.9	1,516	52.3	1,769	53.2
Female	8,120	58.2	4,474	54.4	4,428	54.0	3,562	52.1	2,454	52.1	1,441	48.7	1,559	46.8
Age, years														
65–69	3,782	27.1	1,877	22.8	2,246	27.4	2,045	29.9	1,420	30.1	925	31.3	1,111	33.4
70–74	3,966	28.4	2,275	27.7	2,427	29.6	2,155	31.5	1,619	34.4	1,018	34.4	1,153	34.7
75–79	3,028	21.7	2,053	25.0	1,960	23.9	1,589	23.2	1,036	22.0	653	22.1	711	21.4
≥80	3,177	22.8	2,023	25.6	1,564	19.1	1,053	15.4	636	15.4	361	12.2	353	10.6
Educational attainment, years														
≤9	8,183	58.7	4,698	57.1	3,991	48.7	2,815	41.1	1,737	36.9	971	32.8	807	24.3
10–12	3,859	27.7	2,256	27.4	2,771	33.8	2,548	37.2	1,828	38.8	1,141	38.6	1,394	41.9
≥13	1,520	10.9	984	12.0	1,248	15.2	1,359	19.9	1,088	23.1	804	27.2	1,086	32.6
Other/missing	391	2.8	290	3.5	187	2.3	120	1.8	58	1.2	41	1.4	41	1.2
Annual income, Japanese yen														
<2.00 million	6,283	45.0	3,605	43.8	3,348	40.8	2,609	38.1	1,664	35.3	1,009	34.1	1,009	30.3
2.00–3.99 million	3,629	26.0	2,127	25.9	2,580	31.5	2,483	36.3	1,890	40.1	1,208	40.9	1,555	46.7
≥4.00 million	1,057	7.6	621	7.6	764	9.3	718	10.5	547	11.6	402	13.6	461	13.9
Missing	2,984	21.4	1,875	22.8	1,505	18.4	1,032	15.1	610	13.0	338	11.4	303	9.1
Employment status														
Working	3,187	22.8	1,723	20.9	1,752	21.4	1,438	21.0	941	20.0	554	18.7	596	17.9
Retired	6,573	47.1	4,032	49.0	4,409	53.8	4,025	58.8	2,928	62.2	1,914	64.7	2,277	68.4
Never worked	1,933	13.9	1,052	12.8	970	11.8	695	10.2	425	9.0	268	9.1	248	7.5
Missing	2,260	16.2	1,421	17.3	1,066	13.0	684	10.0	417	8.9	221	7.5	207	6.2
Living situation														
Live alone	1,640	11.8	1,075	13.1	1,120	13.7	808	11.8	541	11.5	353	11.9	404	12.1
Live with others	12,036	86.3	6,981	84.8	6,954	84.8	5,960	87.1	4,133	87.7	2,578	87.2	2,899	87.1
Missing	277	2.0	175	2.1	123	1.5	74	1.1	37	0.79	26	0.88	25	0.75
Marital status														
Married	9,290	66.6	5,509	67.0	5,784	70.6	5,002	73.1	3,542	75.2	2,255	76.3	2,584	77.6
Widowed	3,424	24.5	1,979	24.1	1,804	22.0	1,406	20.6	906	19.2	554	18.7	581	17.5
Divorced	539	3.9	280	3.4	269	3.3	201	2.9	129	2.7	73	2.5	83	2.49
Not married	291	2.1	176	2.14	167	2.0	123	1.8	82	1.7	47	1.6	55	1.7
Other/missing	409	2.9	284	3.5	173	2.1	110	1.6	52	1.1	28	1.0	25	0.75
Smoking status														
Non-smoker	7,824	56.1	4,553	55.3	4,651	56.7	3,908	57.1	2,666	56.6	1,660	56.1	1,841	55.3
Ex-smoker	3,334	23.9	2,007	24.4	2,203	26.9	1,937	28.3	1,461	31.0	956	3.3	1,140	34.3
Smoker	1,695	12.2	1,010	12.3	863	10.5	683	10.0	403	8.6	229	7.7	257	7.7
Missing	1,100	7.9	658	8.0	480	5.9	314	4.6	181	3.8	112	3.8	90	2.7
Alcohol intake														
Non-drinker	9,264	66.4	5,325	64.7	5,111	62.4	3,984	58.2	2,689	57.1	1,548	52.4	1,608	48.3
Ex-drinker	529	3.8	281	3.4	311	3.8	213	3.1	139	3.0	90	3.0	101	3.0
Drinker	3,862	27.7	2,408	29.3	2,618	31.9	2,543	37.2	1,827	38.8	1,279	43.3	1,601	48.1
Missing	298	2.1	214	2.6	157	1.9	102	1.5	56	1.4	40	1.4	18	0.54
BMI, kg/m^2^														
<18.5	1,164	8.3	652	7.9	578	7.1	425	6.2	262	5.6	165	5.6	163	4.9
18.5–24.9	8,921	63.9	5,361	65.2	5,562	67.9	4,832	70.6	3,443	73.1	2,168	73.3	2,489	74.8
25.0–29.9	2,685	19.2	1,576	19.2	1,560	19.0	1,304	19.1	831	17.6	527	17.8	581	17.5
≥30.0	329	2.4	188	2.3	180	2.2	112	1.6	64	1.4	36	1.2	34	1.0
Missing	854	6.1	451	5.5	317	3.9	169	2.5	111	2.4	61	2.1	61	1.8
IADL														
Mean	10.7		11.2		11.7		12.0		12.2		12.4		12.5	
Depressive symptoms														
Non-depressed (GDS <5)	7,056	50.6	4,501	54.7	5,046	61.6	4,624	67.6	3,299	70.0	2,195	74.2	2,621	78.8
Depressed (GDS ≥5)	4,454	31.9	2,190	26.6	1,778	21.7	1,223	17.9	763	16.2	366	12.4	295	8.9
Missing	2,443	17.5	1,537	18.7	1,373	16.8	995	14.5	649	13.8	396	13.4	412	12.4
Cognitive complaints														
No	7,968	57.1	4,831	58.7	5,340	65.2	4,601	67.3	3,344	71.0	2,116	71.6	2,476	74.4
Yes	5,706	40.9	3,195	38.8	2,697	32.9	2,150	31.4	1,303	27.7	812	27.5	820	24.6
Missing	279	2.0	202	2.5	160	2.0	91	1.3	64	1.4	29	1.0	32	1.0
Self-rated health														
Very good	1,143	8.2	805	9.8	814	9.9	909	13.3	688	14.6	510	17.3	738	22.2
Good	9,022	64.7	5,498	66.8	5,826	71.1	4,955	72.4	3,425	72.7	2,095	70.9	2,302	69.2
Poor	3,055	21.9	1,573	19.1	1,309	16.0	818	12.0	529	11.2	290	9.8	237	7.1
Very poor	549	3.9	242	2.9	152	1.4	94	1.4	41	0.9	33	1.1	27	0.81
Missing	184	1.3	110	1.3	96	1.0	66	1.0	28	0.6	29	1.0	24	0.72
Self-reported disease diagnoses														
Cancer (yes)	619	4.4	347	4.2	353	4.3	276	4.0	199	4.2	138	4.7	131	3.9
Heart disease (yes)	1,730	12.4	994	12.1	937	11.4	745	10.9	545	11.6	322	10.9	365	11.0
Stroke (yes)	181	1.3	114	1.4	95	1.16	72	1.1	42	0.89	27	0.91	42	1.3
Diabetes mellitus (yes)	1,802	12.9	1,027	12.5	1,000	12.2	839	12.3	532	11.3	376	12.7	374	11.2
Respiratory disease (yes)	554	4.0	296	3.6	300	3.7	201	2.9	148	3.1	91	3.1	84	2.5
Others (yes)	9,563	68.5	5,810	70.6	5,766	70.3	4,715	68.9	3,206	68.1	2,003	67.7	2,142	64.4
Missing	248	1.8	134	1.6	140	1.7	92	1.3	44	0.93	27	0.91	35	1.1
Frequency of meet friends														
Once or more/week	5,847	41.9	4,167	50.6	4,483	54.7	4,131	60.4	2,958	62.8	1,919	64.9	2,335	70.2
Once or twice/month	2,649	19.0	1,585	19.3	1,689	20.6	1,336	19.5	932	19.8	601	20.3	555	16.7
Rarely	4,586	32.9	2,001	24.3	1,669	20.4	1,188	17.4	706	19.8	370	12.5	379	11.4
Missing	871	6.2	475	5.8	356	4.3	187	2.7	115	15.0	67	2.3	59	1.8
Number of meet friends														
≤5/month	8,202	58.8	4,308	52.4	3,799	46.4	2,612	38.2	1,580	33.5	845	28.6	787	23.7
≥6/month	4,620	33.1	3,295	40.1	4,027	49.1	4,073	59.5	3,040	64.5	2,071	70.0	2,498	75.1
Missing	1,131	8.1	625	7.6	371	4.5	157	2.3	91	1.9	41	1.4	43	1.3
Receive emotional support														
Yes	12,187	87.3	7,236	87.9	7,545	92.1	6,435	94.1	4,471	94.9	2,805	94.9	3,201	96.2
No	1,103	7.9	598	7.3	412	5.0	243	3.6	142	3.0	110	3.7	90	2.7
Missing	663	4.8	394	4.8	240	2.9	164	2.4	98	2.1	42	1.4	37	1.1
Receive instrumental support														
Yes	12,547	89.9	7,464	90.7	7,619	92.9	6,475	94.6	4,471	94.9	2,824	95.5	3,193	95.9
No	846	6.1	437	5.3	384	4.7	237	3.5	159	3.4	95	3.2	87	2.6
Missing	560	4.0	327	4.0	194	2.4	130	1.9	81	1.7	38	1.3	48	1.4

BMI, body mass index; GDS, geriatric depression scale; IADL, instrumental activities of daily living.

Table 2 shows the association of the total number of leisure activities with mortality. There was a statistically significant inverse relationship between the total number of leisure activities and mortality (*P* for linear trend <0.001). When we modeled the total number of leisure activities as a linear variable, the HRs of all-cause mortality were 0.87 (95% confidence interval [CI], 0.86–0.89) in model 1, 0.93 (95% CI, 0.92–0.95) in model 2, and 0.93 (95% CI, 0.92–0.95) in model 3. The E-values for the analyses of the association between the total number of leisure activities and all-cause mortality were calculated; *E* = 1.55 (model 1), *E* = 1.35 (model 2), and *E* = 1.34 (model 3), which are shown in eTable 2.

**Table 2.** Association of the total number of leisure activities with all-cause mortality in older Japanese adults (*n* = 48,216), Japan, 2010–2016

Total number of leisure activities	No. of deaths	Model 1^a^			Model 2^b^			Model 3^c^		
		
		HR	95% CI	*P* for trend	HR	95% CI	*P* for trend	HR	95% CI	*P* for trend
Total number of leisure activities (continuous)		0.87	0.86, 0.89	<0.001	0.93	0.92, 0.95	<0.001	0.93	0.92, 0.95	<0.001

Total number of leisure activities (categorical)				<0.001			<0.001			<0.001
0	2,077	1.00	Referent		1.00	Referent		1.00	Referent	
1	1,180	0.87	0.81, 0.93		0.97	0.90, 1.04		0.97	0.90, 1.04	
2	921	0.74	0.68, 0.80		0.90	0.83, 0.97		0.90	0.83, 0.98	
3	630	0.64	0.58, 0.70		0.83	0.75, 0.91		0.83	0.76, 0.91	
4	363	0.55	0.50, 0.62		0.74	0.66, 0.83		0.75	0.66, 0.84	
5	208	0.50	0.44, 0.58		0.69	0.60, 0.80		0.70	0.60, 0.81	
6–17	196	0.42	0.36, 0.49		0.61	0.52, 0.71		0.61	0.53, 0.72	

CI, confidence interval; HR, hazard ratio.

^a^Cox proportional hazards regression analysis; adjusted for sex, age, education, income, and employment status.

^b^Cox proportional hazards regression analysis; additionally adjusted for living situation, marital status, smoking status, alcohol intake, body mass index, instrumental activities of daily living, depressive symptoms, cognitive complaints, self-rated health status, and chronic diseases (cancer, heart disease, stroke, diabetes mellitus, respiratory disease, and other diseases).

^c^Cox proportional hazards regression analysis; additionally adjusted for frequency of meet friends, number of friends, emotional social support (received), and instrumental social support (received).

Table 3 shows the association of the types and the number of leisure activities in terms of physical activity involvement (“physically-active leisure activities,” “cultural leisure activities,” and “other leisure activities”) with mortality. Both “physically-active leisure activities” and “other leisure activities” were associated with a lower risk of mortality (*P* for linear trend <0.001), whereas there was no significant association between engagement in “cultural leisure activities” and lower mortality in the adjusted models (*P* for linear trend = 0.717 in model 2, 0.827 in model 3).

**Table 3.** Association of the types and the number of leisure activities with all-cause mortality in older Japanese adults (*n* = 48,216), Japan, 2010–2016

Type and the number of leisure activities	No. of deaths	Model 1^a^			Model 2^b^			Model 3^c^		
		
		HR	95% CI	*P* for trend	HR	95% CI	*P* for trend	HR	95% CI	*P* for trend
Physically-active leisure activities				<0.001			<0.001			<0.001
0	3,168	1.00	Referent		1.00	Referent		1.00	Referent	
1	1,403	0.80	0.75, 0.85		0.91	0.85, 0.97		0.91	0.85, 0.97	
2–8	1,004	0.65	0.60, 0.70		0.80	0.74, 0.86		0.80	0.74, 0.86	
Cultural leisure activities				0.026			0.717			0.827
0	3,931	1.00	Referent		1.00	Referent		1.00	Referent	
1	1,099	0.93	0.86, 0.99		0.97	0.90, 1.04		0.97	0.90, 1.04	
2–8	545	0.92	0.84, 1.01		0.99	0.90, 1.09		1.00	0.90, 1.10	
Other leisure activities^d^				<0.001			<0.001			<0.001
0	3,663	1.00	Referent		1.00	Referent		1.00	Referent	
1	1,401	0.86	0.81, 0.92		0.94	0.88, 1.00		0.94	0.88, 1.00	
2–6	511	0.66	0.60, 0.73		0.75	0.68, 0.83		0.76	0.69, 0.84	

CI, confidence interval; HR, hazard ratio.

^a^Cox proportional hazards regression analysis; adjusted for sex, age, education, income, and employment status.

^b^Cox proportional hazards regression analysis; additionally adjusted for living situation, marital status, smoking status, alcohol intake, body mass index, instrumental activities of daily living, depressive symptoms, cognitive complaints, self-rated health status, and chronic diseases (cancer, heart disease, stroke, diabetes mellitus, respiratory disease, and other diseases).

^c^Cox proportional hazards regression analysis; additionally adjusted for frequency of meet friends, number of friends, emotional social support (received), and instrumental social support (received).

^d^Includes PC, karaoke, traveling, fishing, pachinko, and other.

Another analysis, shown in Table 4, examined the association of the types and the number of leisure activities in terms of group activity involvement (“group-based leisure activities,” “solitary leisure activities,” and “others”) with mortality. “Solitary leisure activities” were not associated with significantly lower mortality (*P* for linear trend = 0.006 in model 1, 0.259 in model 2, 0.326 in model 3), whereas “group-based leisure activities” showed a significant dose-response relationship with a lower risk of mortality (*P* for linear trend <0.001). “Others,” which are somewhere in between “group” and “solitary,” were partly associated with a significantly reduced mortality risk because engagement in two or more “others” turned out to lower the risk of all-cause mortality; HR 0.66 (95% CI, 0.61–0.70) in model 1, HR 0.82 (95% CI, 0.76–0.88) in model 2, HR 0.82 (95% CI, 0.76–0.88) in model 3.

**Table 4.** Association of the types and the number of leisure activities with all-cause mortality in older Japanese adults (*n* = 48,216), Japan, 2010–2016

Type and the number of leisure activities	No. of deaths	Model 1^a^			Model 2^b^			Model 3^c^		
		
		HR	95% CI	*P* for trend	HR	95% CI	*P* for trend	HR	95% CI	*P* for trend
Group-based leisure activities				<0.001			<0.001			<0.001
0	4,247	1.00	Referent		1.00	Referent		1.00	Referent	
1	1,006	0.79	0.74, 0.85		0.87	0.81, 0.93		0.87	0.81, 0.94	
2–5	322	0.73	0.65, 0.82		0.85	0.75, 0.95		0.85	0.76, 0.96	
Solitary leisure activities				0.006			0.259			0.326
0	3,836	1.00	Referent		1.00	Referent		1.00	Referent	
1	1,212	0.93	0.87, 0.99		0.96	0.89, 1.02		0.96	0.90, 1.03	
2–7	527	0.90	0.81, 0.99		0.95	0.86, 1.05		0.96	0.87, 1.06	
Others^d^				<0.001			<0.001			<0.001
0	2,882	1.00	Referent		1.00	Referent		1.00	Referent	
1	1,420	0.85	0.80, 0.91		0.95	0.89, 1.02		0.95	0.89, 1.02	
2–9	1,273	0.66	0.61, 0.70		0.82	0.76, 0.88		0.82	0.76, 0.88	

CI, confidence interval; HR, hazard ratio.

^a^Cox proportional hazards regression analysis; adjusted for sex, age, education, income, and employment status.

^b^Cox proportional hazards regression analysis; additionally adjusted for living situation, marital status, smoking status, alcohol intake, body mass index, instrumental activities of daily living, depressive symptoms, cognitive complaints, self-rated health status, and chronic diseases (cancer, heart disease, stroke, diabetes mellitus, respiratory disease, and other diseases).

^c^Cox proportional hazards regression analysis; additionally adjusted for frequency of meet friends, number of friends, emotional social support (received), and instrumental social support (received).

^d^Includes exercise/Tai Chi, walking/jogging, tea ceremony/flower arrangement, photography, gardening, growing crops, traveling, hiking, and other.

Next, we repeated the analyses excluding the deaths occurring within the first one, two, and three years of follow-up in order to address reverse causality (ie, illness symptoms affecting engagement in leisure activity). The total number of leisure activities, “physically-active leisure activities,” and “group-based leisure activities” remained statistically significantly associated with reduced mortality hazard.

We conducted two sets of sensitivity analyses. With the former sensitivity analysis, we found that engagement in “sedentary leisure activities” was not associated with a reduced risk of mortality. After the second sensitivity analysis, it turned out that engagement in “non-physically-active solitary leisure activities” was not associated with a significantly lower risk of mortality, which was in line with the two results above. Data of these sensitivity analyses are available from authors on request.

eTable 3 shows the association of each leisure activity with all-cause mortality (simultaneously mutually adjusted). Some activities such as golf, exercise/Tai Chi, dancing, and craft were independently associated with lower mortality, whereas others such as calligraphy, photography, gardening, and fishing were not.

## DISCUSSION

In the present study, we assessed the association of engagement in leisure activities with all-cause mortality. We found a dose-response association between the total number of leisure activities and all-cause mortality, which is in line with the previous studies.^5^^,^^7^ One possible mechanism is related to psychological aspects of engagement in leisure activities. It has been reported that leisure participation contributes to higher subjective well-being,^44^ and in turn, subjective well-being is associated with a lower risk of mortality.^45^ But more plausibly, the larger the total number of leisure activities that individuals engage in, the higher the likelihood that they are engaged in effective activities for preventing death, such as “physically-active leisure activities” or “group-based leisure activities.”

We also found a dose-response association between engagement in “physically-active leisure activities” and lower all-cause mortality risk. Individuals who engage in a greater number of “physically-active leisure activities” will tend to be more physically active. The observed relationship could be explained by the dose-response protective association of physical activity with mortality which has been widely reported in many prior studies.^46^^,^^47^

Engagement in “group-based leisure activities” was similarly associated with a lower risk of mortality in a dose-response fashion. However, the pattern of decreasing mortality risk was not as clear as that seen in “physically-active leisure activities.” What mattered for lowering the risk of mortality appeared to be whether individuals are engaged in at least one group-based leisure activity or not; additional engagement did not lower the risk further. Previous studies have found that stronger social relationships are associated with a reduced risk of mortality,^25^ which could account for the correlation between “group-based leisure activities” and lower mortality.

In contrast, we did not find a significant association between engagement in “cultural leisure activities” and all-cause mortality. Partially, this is consistent with a previous study that suggested that making music and reading books or periodicals were not associated with a reduced risk of mortality.^18^ However, another study among Finnish employees, aged less than 65 years at the entry to the study, showed that reading and studying were associated with lowered mortality.^19^ The inconsistency may be explained by the difference in the study population. The present study investigated older Japanese adults, whereas the previous research focused on Finnish industrial employees.^19^ As our sensitivity analyses showed that engagement in “non-physically-active solitary leisure activities” was not associated with a lower risk of mortality, solitary cultural activities might have different associations with mortality depending on the current employment status. For the working generation, engaging with solitary cultural activities may be an indicator that they can afford to enjoy life and a high level of health consciousness. Working populations, who are enjoying solitary cultural activities, could be associated with their improved health behavior or health literacy, which might lead to a reduced risk of mortality.^48^

Besides, the present study showed that there was no significant association between participation in “solitary leisure activities” and all-cause mortality. In other words, engaging in solitary activities may have canceled some of the beneficial effects of participation in leisure activities. Loneliness, social isolation, and a low level of social engagement have been reported to be associated with increased mortality.^25^^,^^49^ Furthermore, the result is consistent with a previous study, which showed that increased frequency of exercise with other people was associated with better subjective health status among the elderly Japanese population.^50^

E-values were calculated to assess the robustness of the observed associations to unmeasured confounding. For example, as noted in eTable 2, the observed hazard ratio of 0.93 could be explained away by an unmeasured confounder that was associated with both the total number of leisure activities and mortality by a risk ratio of 1.35-fold each. Such potential unmeasured confounder may include physical environments,^51^ childhood socioeconomic status,^42^ or personality.^52^

Finally, our sensitivity analysis indicated that engagement in “sedentary leisure activities” was also not associated with all-cause mortality. This is inconsistent with the findings of the previous studies,^12^^–^^14^ in which sedentary behavior, measured as watching television, using computers, and sitting reading, showed a significant positive association with mortality. A possible explanation for the inconsistent results is that our sedentary leisure activities primarily involved cognitive or cultural activities, which are linked with beneficial health outcomes.^1^^,^^15^^–^^20^

There are some limitations to the present study. The first limitation concerns endogeneity. We cannot rule out reverse causation, even with longitudinal data. For example, more energetic individuals will tend to be engaged in a greater variety of leisure activities. Hence, the total number of leisure activities can be just a marker for vitality (which is a predictor of living longer). Second, we did not assess the intensity or frequency of engagement in leisure activities. Some people may report “walking/jogging” as their leisure activities, but they might do it only once a month. Third, we did not assess cause-specific mortality. Therefore, the mechanisms of how engagement in leisure activities reduces the risk of mortality are unclear. Fourth, our classifications of different leisure activities might be imprecise. For example, “playing musical instruments” and “fishing” are grouped under “solitary leisure activities,” but some people might always play musical instruments or go fishing with their friends. Finally, the generalizability of the results might be weak because the present analyses used data from participants who reported no disability in activities of daily living and responded to the questions regarding leisure activities and instrumental activities of daily living. The analytic samples were younger, had higher educational attainment, and had a higher income (eTable 4). Besides, according to the Cabinet Office of the Japanese Government, a nationally representative sample showed 11.1% of elderly males and 20.3% of elderly females lived alone in 2010,^53^ and among the analytic sample, 7.3% of males and 16.6% of females answered they lived alone.

In conclusion, we found that engagement in “physically-active leisure activities” and “group-based leisure activities” were significantly associated with a lower risk of all-cause mortality among older Japanese adults. Further research in this field is needed considering the intensity/frequency of engagement, the causes of mortality, and other potential confounding factors, but our findings suggest that it might be helpful to encourage elderly people to engage in physically-active and social forms of leisure activities in order to promote healthy aging. We recommend that policymakers consider whether the intervention/implementation would improve physical activeness and in-person engagement among the community members.

## APPENDIX A. SUPPLEMENTARY DATA

eTable 1. Baseline characteristics of older Japanese male (n = 22,178) and female (n = 26,038) participants (n = 48,216) who were 65 years of age or older, Japan, 2010–2016

eTable 2. Robustness to unmeasured confounding (E-Values^a^) for assessing the causal associations between engagement in leisure activities and all-cause mortality in older Japanese adults (n = 48,216), Japan, 2010–2016

eTable 3. Association of each leisure activity with all-cause mortality in older Japanese adults (n = 48,216), Japan, 2010–2016

eTable 4. Baseline characteristics of analytic sample (n = 48,216) and non-analytic sample (n = 6,321) who were 65 years of age or older, Japan, 2010–2016
