# Supplementary material for: Prospective Study of Engagement in Leisure Activities and All-Cause Mortality Among Older Japanese Adults
Source: J Epidemiol. 2022 Jun 5;32(6):245–53. doi: 10.2188/jea.JE20200427 (PMC9086310; doi:10.2188/jea.JE20200427)
Supplement: Supplementary file 1 [file je-32-245-s001.pdf]

eTable 1. Baseline characteristics of older Japanese male (n=22,178) and female (n=26,038) participants (n=48,216) who were 65 years of age or older, Japan, 2010–2016

Characteristic	Male (n=22,178)		Female (n=26,038)	
	N	%	N	%
Death	3,519	15.9	2,056	7.9
Age, years				
65–69	6,356	28.7	7,050	27.1
70–74	6,735	30.4	7,878	30.3
75–79	5,033	22.7	5,997	23.0
≥80	4,054	18.3	5,113	19.6
Total number of leisure activities				
0	5,833	26.3	8,120	31.2
1	3,754	16.9	4,474	17.2
2	3,769	17.0	4,428	17.0
3	3,280	14.8	3,562	13.7
4	2,257	10.2	2,454	9.4
5	1,516	6.8	1,441	5.5
6–17	1,769	8.0	1,559	6.0
Type and the number of leisure activities				
Physically-active leisure activities				
0	9,839	44.4	12,500	48.0
1	6,224	28.1	6,764	26.0
2–8	6,115	27.6	6,774	26.0
Cultural leisure activities				
0	14,482	65.3	16,263	62.5
1	5,159	23.3	6,095	23.4
2–8	2,537	11.4	3,680	14.1
Other leisure activities[a]				
0	10,823	48.8	15,944	61.2
1	6,670	30.1	7,361	28.3
2–6	4,685	21.1	2,733	10.5
Group-based leisure activities				
0	14,382	64.8	19,549	75.1
1	5,654	25.5	4,995	19.2
2–5	2,142	9.7	1,494	5.7
Solitary leisure activities				

0	12,871	58.0	16,827	64.6
1	6,188	27.9	6,095	23.4
2–7	3,119	14.1	3,116	12.0
Others[b]				
0	8,921	40.2	11,006	42.3
1	5,669	25.6	6,307	24.2
2–9	7,588	34.2	8,725	33.5
Educational attainment, years				
≤9	9,874	44.5	13,328	51.2
10–12	7,008	31.6	8,789	33.8
≥13	4,913	22.2	3,176	12.2
Other/missing	383	1.7	745	2.9
Annual income, Japanese yen				
<2.00 million	9,084	41.0	10,443	40.1
2.00–3.99 million	8,148	36.7	7,324	28.1
≥4.00 million	2,293	10.3	2,277	8.7
Missing	2,653	12.0	5,994	23.0
Employment status				
Working	6,190	27.9	4,001	15.4
Retired	13,492	60.8	12,666	48.6
Never worked	929	4.2	4,662	17.9
Missing	1,567	7.1	4,709	18.1
Living situation				
Live alone	1,613	7.3	4,328	16.6
Live with others	20,220	91.2	21,318	81.9
Missing	345	1.6	392	1.5
Marital status				
Married	18,984	85.6	14,982	57.5
Widowed	1,881	8.5	8,773	33.7
Divorced	572	2.6	1,002	3.8
Not married	332	1.5	609	2.3
Other/missing	409	1.8	672	2.6
Smoking status				
Non-smoker	5,533	24.9	21,570	82.8
Ex-smoker	11,767	53.1	1,271	4.9
Smoker	4,327	19.5	813	3.1

Missing	551	2.5	2,384	9.2
Alcohol intake				
Non-drinker	8,159	36.8	21,370	82.1
Ex-drinker	1,409	6.4	255	1.0
Drinker	12,384	55.8	3,754	14.4
Missing	226	1.0	659	2.5
BMI, kg/m^2				
<18.5	1,211	5.5	2,198	8.4
18.5–24.9	15,476	69.8	17,300	66.4
25.0–29.9	4,467	20.1	4,597	17.7
≥30.0	328	1.5	615	2.4
Missing	696	3.1	1,328	5.1
IADL				
Mean	11.3		11.7	
Depressive symptoms				
Non-depressed (GDS <5)	14,026	63.2	15,316	58.8
Depressed (GDS ≥5)	5,401	24.4	5,668	21.8
Missing	2,751	12.4	5,054	19.4
Cognitive complaints				
No	13,936	62.8	16,740	64.3
Yes	7,962	35.9	8,721	33.5
Missing	280	1.3	577	2.2
Self-rated health				
Very good	2,786	12.6	2,821	10.8
Good	14,867	67.0	18,256	70.1
Poor	3,722	16.8	4,089	15.7
Very poor	626	2.8	512	2.0
Missing	177	0.80	360	1.4
Self-reported disease diagnoses				
Cancer (yes)	1,294	5.8	769	3.0
Heart disease (yes)	3,197	14.4	2,441	9.4
Stroke (yes)	408	1.8	165	0.63
Diabetes mellitus (yes)	3,349	15.1	2,601	10.0
Respiratory disease (yes)	1,007	4.5	667	2.6
Others (yes)	14,370	64.8	18,835	72.3
Missing	259	1.2	461	1.8

Frequency of meet friends				
Once or more/week	10,282	46.4	15,558	59.8
Once or twice/month	4,508	20.3	4,839	18.6
Rarely	6,664	30.0	4,235	16.3
Missing	724	3.3	1,406	5.4
Number of meet friends				
≤5/month	10,959	49.4	11,174	42.9
≥6/month	10,274	46.3	13,350	51.3
Missing	945	4.3	1,514	5.8
Receive emotional support				
Yes	19,699	88.8	24,181	92.9
No	1,793	8.1	905	3.5
Missing	686	3.1	952	3.7
Receive instrumental support				
Yes	20,728	93.5	23,865	91.7
No	977	4.4	1,268	4.9
Missing	473	2.1	905	3.5
Leisure activity				
Golf	2,329	10.5	252	1.0
Mini golf	2,118	9.6	1,893	7.3
Gate ball	362	1.6	373	1.4
Exercise/Tai Chi	808	3.6	2,778	10.7
Walking/jogging	6,045	27.3	4,958	19.0
Go/Shogi/Mahjong	2,432	11.0	220	0.84
Reading	3,843	17.3	3,824	14.7
PC	3,090	13.9	975	3.7
Playing musical instruments	449	2.0	1,020	3.9
Chorus/folk song	212	1.0	1,166	4.5
Karaoke	2,632	11.9	2,736	10.5
Dancing	306	1.4	1,650	6.3
Haiku/Tanka/Senryu	452	2.0	756	2.9
Calligraphy	557	2.5	1,243	4.8
Tea ceremony/flower arrangement	78	0.35	1,192	4.6
Craft	305	1.4	3,723	14.3
Painting/hand-drawn postcards	636	2.9	1,273	4.9
Photography	2,150	9.7	617	2.4

Gardening	5,653	25.5	7,704	29.6
Growing crops	3,128	14.1	3,620	13.9
Traveling	5,312	24.0	5,701	21.9
Hiking	658	3.0	356	1.4
Fishing	2,474	11.2	100	0.38
Pachinko	1,506	6.8	507	1.9
Other	2,448	11.0	3,188	12.2

BMI, body mass index; GDS, geriatric depression scale; IADL, instrumental activities of daily living.

[a] Includes PC, karaoke, traveling, fishing, pachinko, and other.

[b] Includes exercise/Tai Chi, walking/jogging, tea ceremony/flower arrangement, photography, gardening, growing crops, traveling, hiking, and other.

eTable 2. Robustness to unmeasured confounding (E-Values[a]) for assessing the causal associations between engagement in leisure activities and all-cause mortality in older Japanese adults (n=48,216), Japan, 2010–2016

Analysis No.	Leisure Activities Exposure	Model 1		Model 2		Model 3	
		For Effect Estimate	For CI Limit	For Effect Estimate	For CI Limit	For Effect Estimate	For CI Limit
1	Total number of leisure activities	1.55	1.50	1.35	1.30	1.34	1.29
2	Physically-active leisure activities	1.66	1.57	1.41	1.31	1.40	1.30
	Cultural leisure activities	1.24	1.05	1.09	1.00	1.06	1.00
	Other leisure activities	1.66	1.55	1.48	1.35	1.47	1.34
3	Group-based leisure activities	1.65	1.52	1.42	1.27	1.41	1.26
	Solitary leisure activities	1.27	1.08	1.15	1.00	1.14	1.00
	Others	1.65	1.57	1.42	1.33	1.41	1.32

CI, confidence interval.

[a] See VanderWeele and Ding (43) for the formula for calculating E-values.

eTable 3. Association of each leisure activity with all-cause mortality in older Japanese adults (n=48,216), Japan, 2010–2016

Leisure activity	No. of deaths	Model 1[a]		Model 2[b]		Model 3[c]	
		HR	95% CI	HR	95% CI	HR	95% CI
Golf	178	0.74	0.64, 0.87	0.80	0.69, 0.94	0.80	0.69, 0.94
Mini golf	5,191	0.77	0.69, 0.85	0.86	0.78, 0.96	0.87	0.78, 0.96
Gate ball	120	1.00	0.83, 1.20	1.12	0.93, 1.34	1.12	0.93, 1.35
Exercise/Tai Chi	182	0.69	0.60, 0.81	0.76	0.65, 0.88	0.75	0.65, 0.88
Walking/jogging	936	0.86	0.80, 0.93	0.94	0.87, 1.02	0.94	0.87, 1.02
Go/Shogi/Mahjong	352	1.00	0.90, 1.12	0.98	0.88, 1.10	0.98	0.88, 1.10
Reading	788	1.09	1.00, 1.18	1.11	1.02, 1.20	1.11	1.02, 1.21
PC	273	0.74	0.65, 0.84	0.78	0.68, 0.88	0.78	0.68, 0.89
Playing musical instruments	100	0.86	0.70, 1.05	0.87	0.72, 1.07	0.88	0.72, 1.08
Chorus/folk song	86	0.88	0.71, 1.10	0.96	0.77, 1.19	0.96	0.77, 1.19
Karaoke	489	0.88	0.80, 0.97	0.95	0.87, 1.05	0.95	0.87, 1.05
Dancing	101	0.69	0.57, 0.84	0.77	0.63, 0.94	0.77	0.63, 0.94
Haiku/Tanka/Senryu	131	0.92	0.77, 1.10	0.93	0.77, 1.11	0.93	0.78, 1.11
Calligraphy	187	1.02	0.88, 1.19	1.12	0.96, 1.30	1.12	0.96, 1.30
Tea ceremony/flower arrangement	88	0.98	0.79, 1.22	1.05	0.85, 1.30	1.05	0.85, 1.30
Craft	251	0.82	0.72, 0.93	0.87	0.77, 1.00	0.87	0.77, 1.00
Painting/hand-drawn postcards	158	0.95	0.81, 1.12	1.00	0.85, 1.18	1.00	0.85,

							1.18
Photography	258	0.88	0.77, 1.01	0.90	0.79, 1.03	0.90	0.79, 1.03
Gardening	1,243	0.92	0.86, 0.98	0.98	0.91, 1.05	0.98	0.91, 1.05
Growing crops	636	0.85	0.78, 0.93	0.92	0.84, 1.00	0.92	0.84, 1.00
Traveling	793	0.80	0.74, 0.87	0.90	0.82, 0.97	0.90	0.83, 0.98
Hiking	54	0.72	0.55, 0.95	0.78	0.60, 1.03	0.78	0.60, 1.03
Fishing	298	0.93	0.82, 1.05	0.95	0.84, 1.07	0.94	0.84, 1.06
Pachinko	223	1.10	0.95, 1.25	1.04	0.91, 1.19	1.04	0.91, 1.19
Other	461	0.80	0.73, 0.88	0.85	0.77, 0.93	0.85	0.77, 0.94

CI, confidence interval; HR, hazard ratio.

[a] Cox proportional hazards regression analysis; adjusted for sex, age, education, income, and employment status.

[b] Cox proportional hazards regression analysis; additionally adjusted for living situation, marital status, smoking status, alcohol intake, body mass index, instrumental activities of daily living, depressive symptoms, cognitive complaints, self-rated health status, and chronic diseases (cancer, heart disease, stroke, diabetes mellitus, respiratory disease, and other diseases).

[c] Cox proportional hazards regression analysis; additionally adjusted for frequency of meet friends, number of friends, emotional social support (received), and instrumental social support (received).

eTable 4. Baseline characteristics of analytic sample (n=48,216) and non-analytic sample (n=6,321) who were 65 years of age or older, Japan, 2010–2016

Characteristic	Analytic Sample (n=48,216)		Non-Analytic Sample (n=6,321)	
	N	%	N	%
Deaths	5,575	11.6	1,157	18.3
P-value			<0.05	
Sex				
Male	22,178	46.0	2,968	47.0
Female	26,038	54.0	3,353	53.0
P-value			0.15	
Age, years				
65–69	13,406	27.8	1,351	21.4
70–74	14,613	30.3	1,645	26.0
75–79	11,030	22.9	1,561	24.7
≥80	9,167	19.0	1,764	27.9
P-value			<0.05	
Educational attainment, years				
≤9	23,202	48.1	3,245	51.3
10–12	15,797	32.8	1,725	27.3
≥13	8,089	16.8	944	14.9
Other/missing	1,128	2.3	407	6.4
P-value			<0.05	
Annual income, Japanese yen				
<2.00 million	19,527	40.5	2,512	39.7
2.00–3.99 million	15,472	32.1	1,613	25.5
≥4.00 million	4,570	9.5	545	8.6
Missing	8,647	17.9	1,651	26.1
P-value			<0.05	
Employment status				
Working	10,191	21.1	1,142	18.1
Retired	26,158	54.3	2,923	46.2
Never worked	5,591	11.6	790	12.5
Missing	6,276	13.0	1,466	23.2
P-value			<0.05	
Living situation				
Live alone	5,941	12.3	782	12.4

Live with others	41,538	86.1	5,317	84.1
Missing	737	1.5	222	3.5
P-value			<0.05	
Marital status				
Married	33,966	70.4	4,194	66.4
Widowed	10,654	22.1	1,461	23.1
Divorced	1,574	3.3	194	3.1
Not married	941	2.0	123	1.9
Other/missing	1,081	2.2	349	5.5
P-value			<0.05	
Smoking status				
Non-smoker	27,103	56.2	2,069	32.7
Ex-smoker	13,038	27.0	973	15.4
Smoker	5,140	10.7	412	6.5
Missing	2,935	6.1	2,867	45.4
P-value			<0.05	
Alcohol intake				
Non-drinker	29,529	61.2	2,627	41.6
Ex-drinker	1,664	3.5	163	2.6
Drinker	16,138	33.5	1,004	15.9
Missing	885	1.8	2,527	40.0
P-value			<0.05	
BMI, kg/m^2				
<18.5	3,409	7.1	554	8.8
18.5–24.9	32,776	68.0	3,943	62.4
25.0–29.9	9,064	18.8	1,145	18.1
≥30.0	943	2.0	162	2.6
Missing	2,024	4.2	517	8.2
P-value			<0.05	
IADL (before MI)				
13	19,092	39.6	1,027	16.2
≤12	25,187	52.2	2,205	34.9
Missing	3,937	8.2	3,089	48.9
P-value			<0.05	
Depression symptoms				
Non-depressed (GDS <5)	29,342	60.9	2,989	47.3

Depressed (GDS ≥5)	11,069	23.0	1,700	26.9
Missing	7,805	16.2	1,632	25.8
P-value			<0.05	
Cognitive complaints				
No	30,676	63.6	1,891	29.9
Yes	16,683	34.6	1,889	29.9
Missing	857	1.8	2,541	40.2
P-value			<0.05	
Self-rated health				
Very good	5,607	11.6	547	8.7
Good	33,123	68.7	3,246	51.4
Poor	7,811	16.2	1,320	20.9
Very poor	1,138	2.4	472	7.5
Missing	537	1.1	736	11.6
P-value			<0.05	
Self-reported disease diagnoses				
Cancer (yes)	2,063	4.3	324	5.1
P-value			<0.05	
Heart disease (yes)	5,638	11.7	880	13.9
P-value			<0.05	
Stroke (yes)	573	1.2	157	2.5
P-value			<0.05	
Diabetes mellitus (yes)	5,950	12.3	787	12.5
P-value			<0.05	
Respiratory disease (yes)	1,674	3.5	304	4.8
P-value			<0.05	
Others (yes)	33,205	68.9	4,246	67.2
P-value			<0.05	
Missing	720	1.5	490	7.8
P-value			<0.05	
Frequency of meet friends				
Once or more/week	25,840	53.6	2,273	36.0
Once or twice/month	9,347	19.4	790	12.5
Rarely	10,899	22.6	1,277	20.2
Missing	2,130	4.4	1,981	31.3
P-value			<0.05	

Number of meet friends				
≤5/month	22,133	45.9	2,364	37.4
≥6/month	23,624	49.0	1,842	29.1
Missing	2,459	5.1	2,115	33.5
P-value			<0.05	
Receive emotional support				
Yes	43,880	91.0	4,160	65.8
No	2,698	5.6	371	5.9
Missing	1,638	3.4	1,790	28.3
P-value			<0.05	
Receive instrumental support				
Yes	44,593	92.5	4,264	67.5
No	2,245	4.7	315	5.0
Missing	1,378	2.9	1,742	27.6
P-value			<0.05	

BMI, body mass index; GDS, geriatric depression scale; IADL, instrumental activities of daily living; MI, multiple imputations.

The data were analyzed using the Chi-squared test.
